# Stereotactic Radiosurgery and Ipilimumab Versus Stereotactic Radiosurgery Alone in Melanoma Brain Metastases

**DOI:** 10.7759/cureus.1511

**Published:** 2017-07-25

**Authors:** Steven M Nguyen, Aurelio Castrellon, Oliver Vaidis, Andrew E Johnson

**Affiliations:** 1 Department of Civil Engineering, Carnegie Mellon University; 2 Breast Cancer Center, Memorial Cancer Institute; 3 Department of Mathematics and Statistics, University of South Florida; 4 Department of Computer Science, University of Houston

**Keywords:** stereotactic radiosurgery, ipilimumab, melanoma, brain mets, meta-analysis

## Abstract

Benefits of stereotactic radiosurgery (SRS) have been well established in melanoma brain metastases (MBM). Immunotherapy agents such as ipilimumab (ipi) have recently demonstrated clinical efficacy in advanced disease as well. The theoretical synergistic effects of combining these therapies in MBM have not been explored in detail, however, we conducted a systematic review with meta-analysis of studies that compared combined SRS and ipi versus SRS alone in MBM. Medical Literature Analysis and Retrieval System Online (MEDLINE) and Central databases were used for our literature search, which was conducted by three reviewers. We included studies that examined SRS and ipilimumab compared to SRS alone in MBM. Pertinent results were tabulated in a standardized spreadsheet. Newcastle-Ottawa Scale (NOS) Risk of Bias Assessment and Grading of Recommendations, Assessment, Development and Evaluation (GRADE) method for rating evidence quality were used for qualitative analysis. Review Manager was used for statistical analysis. We identified four cohort studies that compared SRS plus ipi versus SRS alone in MBM. As per the GRADE criteria, we found low-quality evidence for survival benefits associated with combined treatment. Meta-analysis confirmed a significant benefit in survival for SRS and ipilimumab (hazard ratio 0.38, 95% confidence interval 0.28 – 0.52, p < 0.01). There were no significant differences between comparison groups for local control, distant brain control, radiation necrosis, or intracranial bleeding. We conclude that low-quality evidence exists for superior overall survival in MBM treated with SRS and ipilimumab compared to SRS without ipilimumab. There is also no increased risk of radiation necrosis and/or intracranial bleeding with combining radiation and immunotherapy in this setting.

## Introduction and background

In 2016, there were 76,380 estimated new melanoma cases in the United States with a projected 10,130 deaths in patients with melanoma [1]. Brain metastases (BM) occur in up to 30% of all cancer patients [2]. For melanoma, there is a known predilection to spread to the brain; it has been documented as having the highest propensity of all malignant cancers to spread to this site [3-4]. Melanoma accounts for 10% of adult brain metastases (BM) cases as the third leading cause after lung and breast primary cancers [5]. The BM incidence in those with advanced melanoma ranges from 10-74% [6-8]. Due to the aggressive nature of melanoma, those with melanoma brain metastases (MBM) carry high mortality rates (81-95%) and usually die of neurocognitive sequelae [8-9]. Furthermore, melanoma is among the group of known radioresistant cancers [10-11]. The BM lesions are typically treated with surgical resection, stereotactic radiosurgery (SRS), and/or whole-brain radiation therapy (WBRT). Current management recommendations are based on the patients’ general prognosis, in addition to the number, size, and location of brain lesions [12]. SRS is often used as adjuvant therapy in surgical resection of single, accessible tumors. The surgery alone can improve symptomatic burden with BM, however, local control (LC) failure has been reported as high as 59% at two-year follow-up [13]. Post-operative radiosurgery to the tumor bed has demonstrated good LC (72% at 12 months) in the previous retrospective study [14]. SRS alone has also been reported to improve LC when compared to surgical resection in this setting [15]. Advances in stereotactic techniques have made SRS a popular option in the context of MBM and have supplanted WBRT in upfront treatment. Whole-brain radiation therapy (WBRT) (30 Gy, 10 fractions) is generally reserved for large lesions (> 4cm) and patients with heavy intracranial burden [16]. SRS maximum tolerated doses are typically 24 Gy, 18 Gy, and 15 Gy for tumors sized < 2cm, 2-3cm, and 3-4cm, respectively [17].

Major developments in immunotherapies have demonstrated improved survival in the patients with advanced melanoma [18-19]. Ipilimumab is a monoclonal antibody that inhibits cytotoxic T-lymphocyte antigen-4 (CTLA-4), which is one of the several immunological checkpoints targeted by novel immunotherapies. The T-lymphocyte activation, as part of the adaptive immune system, can be attenuated via co-inhibitory surface receptors such as CTLA-4. These receptors are naturally expressed by helper-T cells; they have a stronger affinity for antigen presenting cell B7 ligand than to co-immunostimulatory T-cell CD28 receptors. As a result of CTLA-4 signaling, cytotoxic T-cell activity is inhibited [20]. Immunotherapy research has targeted this process with the rationale that limiting this immunological inhibition will bolster physiological response to cancers. In 2011, the Food and Drug Administration approved ipilimumab for the treatment of patients with unresectable (inoperable) or metastatic melanoma. The drug was approved based on results from a pivotal randomized, double-blind phase three study [19]. Hodi, et al. investigated ipilimumab therapy (n = 137) in comparison to the gp100 peptide cancer vaccine (n = 136) and revealed improved overall survival (OS) in patients receiving ipilimumab [19]. The overall survival for patients receiving ipilimumab alone was 10.1 months compared to 6.4 months in the gp100 alone arm (hazard ratio (HR) 0.68, p = 0.003). Combined therapy (n = 403) had a median OS of 10.0 months [19].

Although radiotherapy has been extensively studied in the context of MBM, there is limited data on its combined usage with immunotherapy agents such as ipilimumab with virtually no prospective results. Given the robust efficacy of novel immunotherapies in advanced neoplastic disease, further exploration of their effects in the brain, in the setting of radiation treatment is warranted. We conducted a systematic review of recent studies that examined stereotactic radiosurgery and ipilimumab treatment in MBM. We aimed to compare clinical outcomes and efficacy between combined treatments and radiation alone.

## Review

Methodsd

Our protocol was registered in PROSPERO register of systematic reviews [21]. We performed a literature search for studies that compared SRS and ipilimumab versus SRS alone in the treatment of MBM. We used MEDLINE and CENTRAL databases as part of our search. Search terms included: "ipilimumab"and "stereotactic" or "radiosurgery" and "melanoma" or "brain" or "metastases". We included randomized controlled trials, non-randomized controlled trials, retrospective cohort studies. Survey reviews, systematic reviews, case reports, and meta-analyses were excluded. Studies that involved advanced melanoma patients without brain metastases were excluded. A standardized spreadsheet was used to organize extracted data. This process was performed by three independent reviewers. We used the GRADE system for rating the quality of evidence [22]. The quality of evidence is assessed for each outcome individually as either high, moderate, low, or very-low based on scoring criteria, which included study type, a risk of bias, inconsistency, indirectness, imprecision, publication bias, effect size, dose response, and residual confounding effects. We utilized the Newcastle-Ottawa Scale (NOS) Risk of Bias Assessment tool for cohort studies which explores bias in selection, comparability, and outcome assessment [23]. Each metric is awarded a maximum of one “star” and tallied for a total maximum score of nine. Higher scores indicate the lower risk of bias. Primary outcomes were OS, LC, and distant brain control (DBC). Secondary outcomes included treatment-related adverse effects. LC was defined as an arrest of tumor progression at the lesion site. DBC was defined as freedom from new intracranial metastatic lesions at sites that were not present for pre-intervention. The follow-up radiological assessment was required. There was significant heterogeneity among chosen studies. Thus, a true meta-analysis (even with attempted bootstrap resampling) was not possible. However, when making the assumption that the distributions of time-to-event (death) were exponential, we could estimate hazard ratios with 95% confidence intervals (CI) for a study’s median survival (MS) results and generate a forest plot. Generic inverse analysis with fixed effects model was used in Review Manager software for our brief meta-analysis [24].

Results

**Figure 1:**
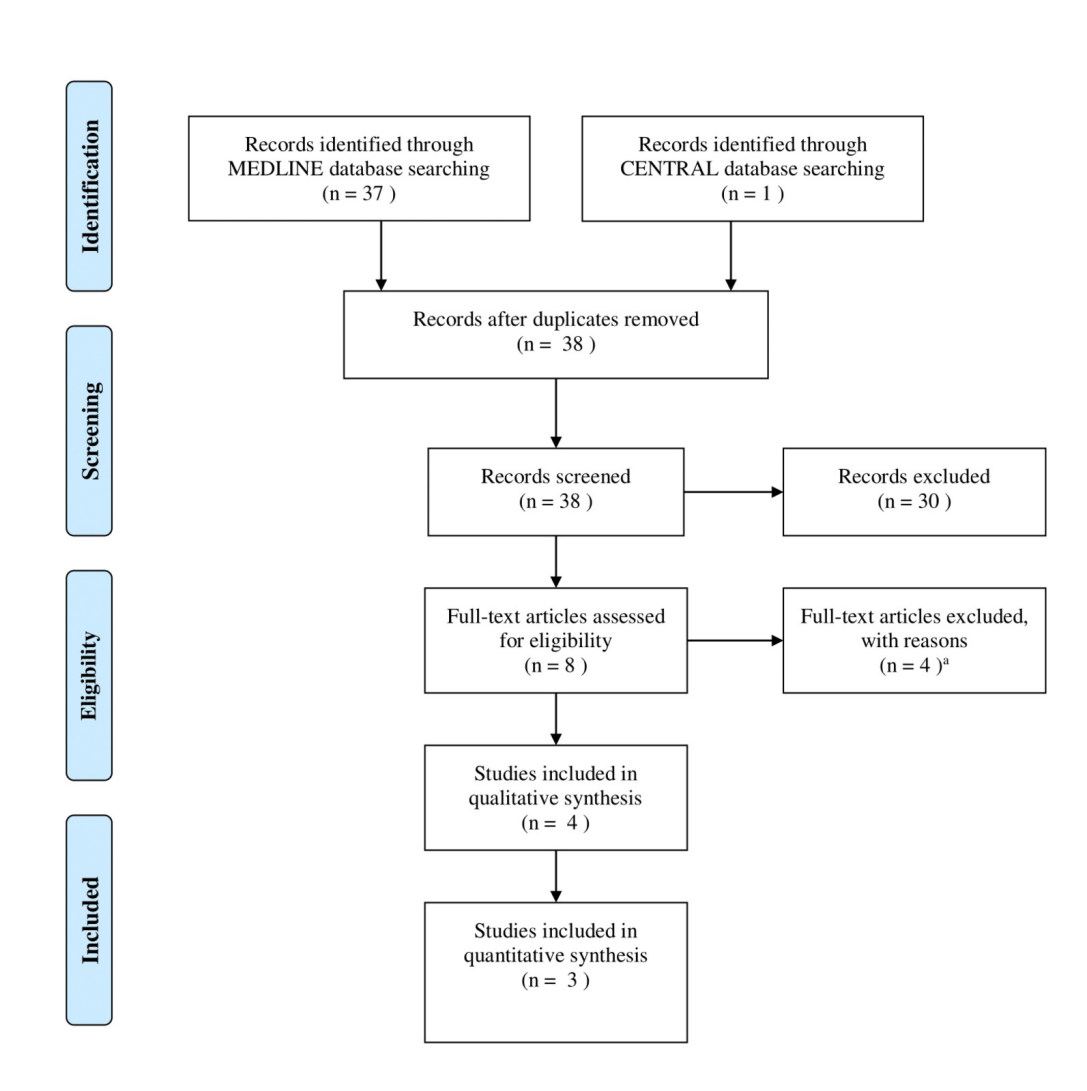
Preferred reporting items for systematic reviews and meta-analyses (PRISMA) flowchart of the literature search and study selection The studies by Tazi, et al., Cohen-Inbar, et al., and Kiess, et al. were excluded. Their study arms were incompatible for our review [25-27]. Silk, et al. abstract was excluded due to results not being reported [28]

*Study and Patient Characteristics:* Thirty-seven publications were identified. Four retrospective studies were eligible for analysis. Our literature search process is illustrated in Figure 1. Tables 1-2-3 summarize relevant outcomes of the selected studies. A retrospective report by Tazi, et al. was excluded as comparison groups were based on whether or not brain metastases were present at the time of ipilimumab administration [25]. No comparisons were made between patients receiving SRS and ipilimumab and those receiving SRS alone. Two other retrospective studies were excluded for similar reasons [26-27]. They compared different timing sequences of ipilimumab with SRS treatment. There was not enough reported data to extrapolate the necessary results for our analysis. A randomized trial by Silk, et al. was also excluded [28]. It was published as an abstract that primarily described an ongoing phase II study with no reported results. For our review, p-value < 0.05 indicated statistical significance.

**Table 1:** Characteristics of stereotactic radiosurgery and ipilimumab therapy studies (2012-2015) selected for analysis. ^a^Silk, et al. included patients receiving WBRT as primary radiotherapy; these patients were excluded for the rest of our review [30]. Abbreviations: SRS = stereotactic radiosurgery; ipi = ipilimumab; RT = radiotherapy; DS-GPA = diagnosis-specific graded prognostic assessment; KPS = Karnofsky performance score; WBRT = whole-brain radiotherapy; fr = fraction; NR = not reported [29-32].

	Patel, et al. [29]	Silk, et al. [30]	Mathew, et al. [31]	Knisely, et al. [32]
Cohort size: SRS plus Ipi	20	17	25	27
Cohort size: SRS alone	34	16	33	50
RT dose^a ^(SRS modality)	15 - 21 Gy, 1 - 5 fr (linear accelerator)	SRS: 14 - 24 Gy, 1 - 5 fr (linear accelerator); WBRT: 30 - 37.5 Gy, 10 - 13 fr	15 - 20 Gy, 1 fr (Gamma Knife)	RT dose NR (Gamma Knife)
Ipi dose	3 mg/kg; concurrent, sequential	3 mg/kg; concurrent, sequential	3, 10 mg/kg; concurrent, sequential	NR; sequential
DS-GPA/KPS	DS-GPA (0-2): 45% vs. 32.4%, p = 0.43	DS-GPA (0-2): 54.5% vs. 54.1%, p = 0.99	Median KPS (range): 90 (80-90) vs. 90 (60-90), p = 0.44	DS-GPA (0-2): 40.7% vs. 64.0%, p = 0.21

**Table 2:** Summary of reported primary outcomes. Outcomes are reported as combined treatment versus stereotactic radiosurgery alone. Abbreviations: mo = months; OS = overall survival; LC = local control; DBC = distant brain control; HR = hazard ratio; CI = confidence interval; NR = not reported; NS = not significant [29-32].

	Patel, et al. [29]	Silk, et al. [30]	Mathew, et al. [31]	Knisely, et al. [32]
Median OS (mo)	8.0 vs. 9.1, HR 1.07, p = 0.84	19.9 vs. 4.0, HR 0.31, p < 0.01	NR	21.3 vs. 4.9; HR 0.48, 95% CI, 0.24 - 0.93, p = 0.03
6-mo OS (%)	79 vs. 69	100 vs. 38	56 vs. 46, p = 0.18	74 vs. 48
12-mo OS (%)	37 vs. 39	83 vs. 32	33 vs. 24, p = 0.18	70 vs. 34
6-mo LC (%)	NR	NR	65 vs. 63, p = 0.55	NR
12-mo LC (%)	71 vs. 92, p = 0.40	NR	42 vs. 45, p = 0.55	NR
6-mo DBC (%)	NR	NR	35 vs. 47, p = 0.48	NR
12-mo DBC (%)	13 vs. 29, p = 0.59	NR	16 vs. 29, p = 0.48	NR

**Table 3:** Summary of reported secondary outcomes. Outcomes are reported as combined treatment versus stereotactic radiosurgery alone. Abbreviations: mo = months; OS = overall survival; LC = local control; DBC = distant brain control; HR = hazard ratio; CI = confidence interval; NR = not reported; NS = not significant [29-32].

	Patel, et al. [29]	Silk, et al. [30]	Mathew, et al. [31]	Knisely, et al. [32]
Radiation necrosis	30.0 vs. 20.9, p = 0.08	0.0 vs. 9.4, p = NS	0.0 vs. 0.0, p = NS	NR
Intracranial hemorrhage	15.0 vs. 14.7, p = 1.00	3.9 vs. 12.5, p = NR	28.0 vs. 30.0, p = NS	NR

**Figure 2:**
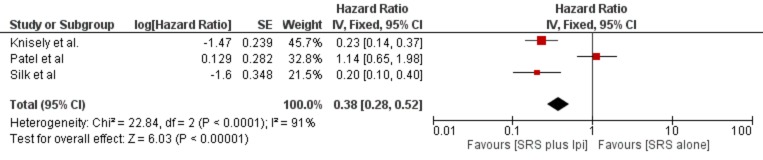
Pooled analysis for median survival Forest plot of hazard ratios and 95% confidence intervals for pooled estimates of Silk, et al., Patel, et al., and Knisely, et al. studies [29-30,32]. Abbreviations: SRS = stereotactic radiosurgery; ipi = ipilimumab; SE = standard error; CI = confidence interval

*Primary and Secondary Outcomes: *Each study [29-32] compared ipilimumab plus SRS and SRS alone in MBM. However, there was noticeable heterogeneity. For example, the studies by Silk, et al. and Knisely, et al. did not report six and 12-month LC and DBC rates [30,32]. Each study reported survival statistics in one form or another, but there was not enough reported data to perform a direct meta-analysis. However, we were able to generate a forest plot for MS outcomes using estimations of HRs with 95% CIs. To accomplish this, we assumed time-to-death demonstrated exponential distribution, which means that the death rate was constant over time. This method allows an approximation of HRs with corresponding 95% CIs from reported MS values; the calculated results are typically closely approximate but not identical to results that would be generated from the raw data. Figure 2 displays our pooled MS analysis based on approximated calculations using the Knisely, et al., Patel, et al., and Silk, et al. results [29-30,32]. For SRS plus ipilimumab compared to SRS alone in MBM, pooled analysis of 222 patients revealed significant benefit in MS (HR 0.38, 95% CI 0.28 – 0.52, p < 0.0001). The Patel, et al. cohorts did not show the significant survival advantage for combination therapy (8.0 vs. 9.1 months, HR 1.07, p = 0.84) [29]. Mathew, et al. did not report MS, however, their analysis showed a non-significant trend towards improved six and 12-month survival rates (56% vs. 46% and 33% vs. 24%, respectively; p = 0.18) [31]. Conversely, Silk, et al. and Knisely, et al. demonstrated a profound survival benefit in their study arms as seen in Table 2 [30,32]. We could only utilize the Patel, et al. and Mathew, et al. studies to report LC and DBC outcomes [29,31]. They found no significant differences in LC or DBC between comparison groups. Secondary outcomes were adverse effects, which primarily were radiation necrosis and intracranial (IC) hemorrhaging as per radiological confirmation. Toxicity profiles were similar between treatment groups as summarized in Table 3. Interestingly, Mathew, et al. reported no cases of radiation necrosis related to therapy in any patient [31].

*Evidence Grade and Risk of Bias Assessment:* Table 4 summarizes our risk of bias assessment for each study using NOS [23]. The Patel, et al., Silk, et al. and Knisely, et al. studies each received a risk of bias score of 6/9 while the Mathew, et al. report scored 4/9 [23,29-32]. Each study received one out of the maximum three “stars” for cohort selection due to selection biases inherent in the retrospective study. Mathew, et al. received zero out of the maximum “two” stars for comparability [31]. The analysis did not further adjust for potential confounders, which the authors mentioned and would introduce comparability bias.

**Table 4:** Risk of bias assessment. The risk of bias assessments was performed using the Newcastle-Ottawa Scale Risk of Bias Assessment tool [23].

	Patel, et al. [29]	Silk, et al. [30]	Mathew, et al. [31]	Knisely, et al. [32]
Representativeness of exposed cohort				
Selection of non-exposed cohort				
Ascertainment of exposure	*	*	*	*
Outcome of interest not present at start of study				
Comparability (max 2)	* *	* *		* *
Assessment of outcome	*	*	*	*
Adequacy of follow-up duration	*	*	*	*
Adequacy of follow-up completeness	*	*	*	*
Total score (max 9)	6/9	6/9	4/9	6/9

Per GRADE methodology, survival outcomes received a “low” evidence quality grade [22]. All other outcomes were rated “very low”. Tables 5-7 summarize our grading. Each outcome assessment was initially rated “low” for their retrospective nature. Each study was further downgraded due to imprecision (sample size lower than optimal information size) and risk of bias. For survival outcomes, we subsequently upgraded the quality due to the large effect of combined therapy on survival. Additionally, the authors mention that these outcomes are potentially affected by residual confounders that would naturally be expected to reduce the demonstrated effect (survival), meaning, combination treatment patients may have received ipilimumab in addition to radiotherapy due to clinician’ judgment that they were “sicker” compared to the SRS-alone group. This plausibility also raised the quality grades. Finally, for any outcome of interest, studies either found a significant benefit for combination therapy, revealed no significant differences between comparison cohorts, or did not report the outcome at all. There were no studies that suggested significant benefit with SRS alone for any outcome.

**Table 5:** Grading of recommendations, assessment, development and evaluation (GRADE) evidence quality ratings for survival outcomes. The quality of evidence assessments was performed using Grading of Recommendations, Assessment, Development and Evaluation (GRADE) system [22]. Abbreviations: SRS = stereotactic radiosurgery.

	Median survival	Six-month survival	12-month survival
Studies demonstrating significant improvement	2/4	2/4	2/4
SRS and Ipilimumab patients	89	89	89
SRS alone patients	133	133	133
Initial quality of evidence	Low (two plus)	Low (two plus)	Low (two plus)
Risk of bias	Serious (-1)	Serious (-1)	Serious (-1)
Inconsistency	Not serious	Not serious	Not serious
Indirectness	Not serious	Not serious	Not serious
Imprecision	Serious (-1)	Serious (-1)	Serious (-1)
Publication bias	Not likely	Not likely	Not likely
Large effect	Large (+1)	Large (+1)	Large (+1)
Dose response	No gradient	No gradient	No gradient
All plausible residual confounding	Would reduce effect (+1)	Would reduce effect (+1)	Would reduce effect (+1)
GRADE overall quality of evidence	Low (two plus)	Low (two plus)	Low (two plus)

**Table 6:** Grading of recommendations, assessment, development and evaluation (GRADE) evidence quality ratings for local and distant brain control outcomes. The quality of evidence assessments was performed using GRADE system [22]. Abbreviations: SRS = stereotactic radiosurgery; DBC = distant brain control.

	Six-month Local control	12-month Local control	6-mo DBC	12-month DBC
Studies demonstrating significant improvement	0/1	0/2	0/1	0/2
SRS and Ipilimumab patients	25	45	25	45
SRS alone patients	33	67	33	67
Initial quality of evidence	Low (two plus)	Low (two plus)	Low (two plus)	Low (two plus)
Risk of bias	Serious (-1)	Serious (-1)	Serious (-1)	Serious (-1)
Inconsistency	Serious (-1)	Serious (-1)	Serious (-1)	Serious (-1)
Indirectness	Not serious	Not serious	Not serious	Not serious
Imprecision	Serious (-1)	Serious (-1)	Serious (-1)	Serious (-1)
Publication bias	Not likely	Not likely	Not likely	Not likely
Large effect	Not likely	Not likely	Not likely	Not likely
Dose response	No gradient	No gradient	No gradient	No gradient
All plausible residual confounding	N/A	N/A	N/A	N/A
GRADE overall quality of evidence	Very Low	Very Low	Very Low	Very Low

**Table 7:** Grading of recommendations, assessment, development and evaluation (GRADE) evidence quality ratings for secondary outcomes. The quality of evidence assessments was performed using grading of recommendations, assessment, development and evaluation (GRADE) system [22]. Abbreviations: SRS = stereotactic radiosurgery.

	Radiation necrosis	Intracranial hemorrhage
Studies demonstrating significant improvement	0/4	0/3
SRS and Ipilimumab patients	89	62
SRS alone patients	133	83
Initial quality of a body of evidence	Low (two plus)	Low (two plus)
Risk of bias	Serious (-1)	Serious (-1)
Inconsistency	Serious (-1)	Serious (-1)
Indirectness	Not serious	Not serious
Imprecision	Serious (-1)	Serious (-1)
Publication bias	Not likely	Not likely
Large effect	Not likely	Not likely
Dose response	No gradient	No gradient
All plausible residual confounding	N/A	N/A
GRADE overall quality of evidence	Very Low	Very Low

Discussion

The studies of interest in this review explore whether radiation and novel immunotherapy agents work synergistically in managing brain lesions [29-32]. There is preclinical and clinical data that support this theory. Ionizing radiation has been shown to increase blood-brain barrier permeability in the preclinical setting [33-34]. Coupled with this phenomenon in addition to the ability of activated T-lymphocytes to freely move across the blood-brain barrier, ipilimumab can further augment tumor-specific cytotoxic response [34-35]. A recent review by Berghoff, et al. [36] discusses the clinical response of lesions without prior irradiation to immunotherapy combined with radiotherapy. This response, called the abscopal effect, is theorized to be the result of radiation-induced antigen release from cell death, which subsequently bolsters T-cell action that is further augmented by immune checkpoint inhibitor therapy [37].

Our analysis revealed a general trend towards improved survival in MBM patients treated with SRS and ipilimumab with a safety profile comparable to those receiving SRS alone. The Silk, et al. and Knisely, et al. studies showed profoundly superior survival for combination therapy [31-32]. The Mathew, et al. report did not find a significant difference in survival outcomes but at least suggest a trend towards benefit (six and 12-month survival: 56% vs. 46% and 33 vs. 24%, respectively, p = 0.18). Although we could not pool LC or DBC results, Mathew, et al. and Patel, et al. found no difference in these outcomes [29,31]. Interestingly, the Patel, et al. cohorts showed no difference in survival. In fact, patients treated only with SRS survived longer (MS 9.1 months vs. 8.0 months, p = 0.84) [29]. When assuming the exponential distribution for time-to-death, pooling the Knisely, et al., Silk, et al., and Patel, et al. cohorts revealed a significant benefit in MS for those receiving combined treatment (HR 0.38, 95% CI, 0.28 – 0.52, p < 0.01) [29-30,32]. However, our output revealed considerable heterogeneity (I-squared = 91%) across included studies. Regarding radiation necrosis and intracranial (IC) bleeding, there was not enough reported data to pool results for quantitative analysis, much like with LC and DBC. However, for studies that did a report on these toxicities, we found that the added ipilimumab therapy did not increase radiation necrosis or IC hemorrhaging incidents. Silk, et al. interestingly showed superior safety profile in combination treatment, but this difference was not significant (radiation necrosis: 0% vs. 9.4%, p = NS; intracranial bleeding: 3.9% vs. 12.5%, p = NR) [30]. Conversely, Patel, et al. found the higher risk of adverse effects in the ipilimumab-treated patients, but this was not statistically significant. Thirty percent of combination therapy patients reported radiation necrosis versus 20.9% in the SRS-alone group (p = 0.08) [29]. Ultimately, it appears that toxicity in SRS-treated MBM patients with added ipilimumab is comparable to those receiving radiation only.

The lack of any randomized prospective data in our search decreased the quality of evidence we found in our analysis. There are inherent limitations and biases introduced in retrospective analysis, which the authors of each study acknowledged. All studies demonstrated minimal to moderate amount of bias risk per the NOS tool [23]. Evidence quality grading demonstrated very-low to low-quality grades for outcomes of interest per GRADE criteria [22]. Each study had small sample sizes ranging from 17 to 27 for groups treated with SRS and ipilimumab and 16 to 50 for SRS alone cohorts, which downgraded the evidence quality. Ultimately, evidence for improved OS in combined treatment is “low quality” using this grading approach. Evidence quality for other outcomes (LC, DBC, toxicity), which did not reveal significant differences across all studies, received a “very low quality” grade.

## Conclusions

The results of our systematic review confirm the need for more prospective data for use of radiation and immunotherapy in melanoma brain metastases (MBM). Although the evidence is low at best, our analysis shows that there are retrospective data that demonstrate promising trends. Combining stereotactic radiosurgery and ipilimumab in melanoma brain metastases can dramatically improve survival rate compared to stereotactic radiosurgery without immunotherapy. There is no increased risk of radiation necrosis and/or intracranial bleeding with combining radiation and immunotherapy in this setting.
